# Mycogenic Silver Nanoparticles: Promising Antimicrobials with Fungistatic Properties

**DOI:** 10.3390/ijms26146639

**Published:** 2025-07-10

**Authors:** Aleksandra Tończyk, Katarzyna Niedziałkowska, Marta Nowak-Lange, Przemysław Bernat, Katarzyna Lisowska

**Affiliations:** 1 Department of Industrial Microbiology and Biotechnology, Faculty of Biology and Environmental Protection, University of Lodz, 12/16 Banacha Street, 90-237 Lodz, Poland; aleksandra.tonczyk@biol.uni.lodz.pl (A.T.); katarzyna.niedzialkowska@biol.uni.lodz.pl (K.N.); marta.nowak@biol.uni.lodz.pl (M.N.-L.); przemyslaw.bernat@biol.uni.lodz.pl (P.B.); 2 The BioMedChem Doctoral School, University of Lodz, Lodz Institutes of Polish Academy of Sciences, 21/23 Matejki Street, 90-237 Lodz, Poland

**Keywords:** silver nanoparticles, biogenic, antifungal, cell membrane, fluidity, permeability, lipidome

## Abstract

The antimicrobial activity of silver nanoparticles (AgNPs) makes them a valuable tool in various industries. Recently, biosynthesis has become the preferred method for nanoparticle synthesis, and among organisms that can be used as AgNP producers, filamentous fungi have attracted the greatest interest. In particular, wood decay fungi are considered promising candidates for AgNP biosynthesis. Biogenic AgNPs have been proven to have strong antibacterial potential and antifungal activity. The aim of this study was to evaluate the antifungal potential of AgNPs synthesized using the brown-rot decay fungus *Gloeophyllum striatum* DSM 9592 against four pathogenic fungal strains: *Candida albicans*, *Malassezia furfur*, *Aspergillus flavus* and *Aspergillus fumigatus*. Moreover, changes in the tested strains’ lipidome and cell membrane properties induced by the presence of AgNPs were investigated. The results revealed that the obtained AgNPs exerted fungistatic activity against all the strains tested. *M. furfur*, with a MIC value of 0.39 μg/mL obtained for all AgNP types, was found to be the most susceptible to the action of AgNPs. The lipidomic analysis revealed that the presence of AgNPs caused an increase in cell membrane fluidity in both *A. flavus* and *C. albicans*, and the mechanisms of response to AgNPs differed between the tested strains.

## 1. Introduction

Silver has been recognized as an effective antimicrobial agent for centuries. Silver nanoparticles (AgNPs), a product of the modern field of science called nanotechnology, are proven to possess similar potential. In general, nanoparticles have a more extensive range of possible applications compared to their bulk materials. This broader applicability also applies to AgNPs, whose scope of application includes medicine, pharmaceuticals and cosmetics, as well as engineering and other technologies [1,2].

Recently, biosynthesis has become the preferred method for nanoparticle synthesis. Biosynthesis provides nanoparticles with the desired properties while simultaneously being more efficient, cost-effective and eco-friendly compared to conventional chemical and physical production methods. In the case of AgNPs, various species have been proven to be able to reduce silver ions, which leads to the formation of nanoparticles [3]. Filamentous fungi are considered the best option for metallic nanoparticle biosynthesis. Their beneficial properties include ease of cultivation and the ability to secrete numerous proteins that act as reducing, capping and stabilizing agents during the production process [4,5]. The number of investigated fungal strains for AgNP synthesis continues to increase [6]. Fungi from the *Aspergillus*, *Penicillium*, *Fusarium* and *Trichoderma* genera are recognized for their ability to synthesize AgNPs [7], and recent studies have proven that other fungal sources, such as *Alternaria* sp. [8], *Rhizoctonia solani*, *Cladosporium cladosporoides* [9] and *Talaromyces funiculosus* [10], can be successfully used in green methods of AgNP production. Species from the wood decay fungi group are interesting yet poorly investigated candidates for AgNP producers. This is mostly due to their ability to produce large amounts of different biologically active compounds [11] that can serve as crucial agents during AgNP synthesis. Moreover, fungi belonging to this group exhibit other properties that could benefit production processes, such as a fast growth rate, resulting in high biomass production, and ease of cultivation [12]. Some reports show that white-rot fungi, e.g., *Trametes* sp., *Ganoderma* sp. and *Phanorochatete* sp., can be used as AgNP sources [13,14]. On the contrary, brown-rot fungi have rarely been researched for this purpose. Given their proven abilities in other biotechnological processes, such as decolorization of dyes [15] or degradation of xenobiotics [16], brown-rot fungi have emerged as another promising option for nanoparticle production. The search for new sources of AgNPs is still of great importance, as biosynthesis processes can vary depending on the fungal species, which produce distinct metabolites affecting the properties of the final products [17]. Our previous work revealed that the brown-rot fungus *Gloeophyllum striatum* is able to reduce silver ions, resulting in AgNP production [18,19].

AgNPs of biological origin are known to possess strong antibacterial potential [7]. It is also proven that AgNPs can work effectively against fungal pathogens, such as *Aspergillus* sp., *Fusarium* sp. and *Candida albicans*. The importance of this property is emphasized by the fact that AgNPs can exert stronger antifungal effects against both human and plant pathogens compared to conventional fungicidal agents [20,21]. The antifungal potential of AgNPs is a result of simultaneous processes caused by exposure to nanoparticles, such as accumulating reactive oxygen species, inducing potassium ion efflux and decreasing the activity of cellular enzymes. AgNPs and silver ions released from the surface of nanoparticles can alter the transcriptome, epigenome and metabolome of fungal cells, negatively influencing their vital functions [22]. Given that pathogenic fungal strains have been reported to develop resistance to commonly used agents [23,24], the complex action of AgNPs against fungi has been gaining great significance.

As fungal infections are one of the most commonly occurring threats to skin health globally [25], evaluating the antifungal activity of *G. striatum*-derived AgNPs was deemed necessary, and four different pathogenic fungal strains were selected for tests. *Candida albicans* is a yeast strain that can be found on mucosal surfaces in healthy humans. However, as an opportunistic pathogen, it can become a cause of infections that affect mucosa, e.g., oral, oropharyngeal or vulvovaginal candidiasis. Moreover, infections caused by *C. albicans* can cross mucosal barriers [26]. *Malassezia furfur* is a yeast-like, commensal microorganism colonizing human skin and can cause a variety of skin diseases, such as dandruff, alopecia or atopic dermatitis. It has also been reported that invasive infections caused by this pathogen are occurring more frequently [27,28]. *Aspergillus fumigatus* and *Aspergillus flavus* belong to a large group of commonly encountered saprophytic filamentous fungi that can be isolated from the air, the soil or materials of plant origin. *Aspergillus* species can be the causal agents of certain superficial and cutaneous mycoses, including onychomycosis and primary cutaneous aspergillosis. Skin infections caused by Aspergilli are most often developed during hospitalization, commonly in infants and patients with immunodeficiencies. Such infections are also a risk factor in surgeries, burns and catheter usage in patients [29,30]. Conventional antifungal drugs, such as clotrimazole, ketoconazole and itraconazole, are commonly provided in topical formulations and administered via spreading or rubbing. The topical delivery of antifungal agents enables their direct access to the target, increases the efficacy of treatment and reduces the risk of developing systemic toxicity [25]. AgNPs with antimicrobial properties can be used in “on-skin” formulations, e.g., in wound care products such as dressings or creams [31]. Consequently, using AgNPs with strong antifungal properties in a topical formulation can be considered beneficial, as both the efficient activity of the agent and the advantages of the topical delivery route would be maintained.

The aim of this study was to evaluate the antifungal activity of AgNPs synthesized using the *G. striatum* DSM 9592 strain against four pathogenic fungal strains, namely, *Candida albicans* ATCC 10231, *Malassezia furfur* DSM 6170, *Aspergillus flavus* ATCC 9643 and *Aspergillus fumigatus* ATCC 204305, in order to determine their possible application potential in combating fungal infections. Moreover, the possible mechanisms of action of AgNPs were explored by performing a lipidomic analysis to identify changes in phospholipid profiles and evaluating changes in cell membrane fluidity and permeability in the presence of AgNPs. To the best of our knowledge, this is the first investigation of the antifungal properties exhibited by mycogenic AgNPs synthesized using a brown-rot fungus.

## 2. Results

### 2.1. Evaluation of the Antifungal Activity of Mycogenic AgNPs

**Figure 1:**
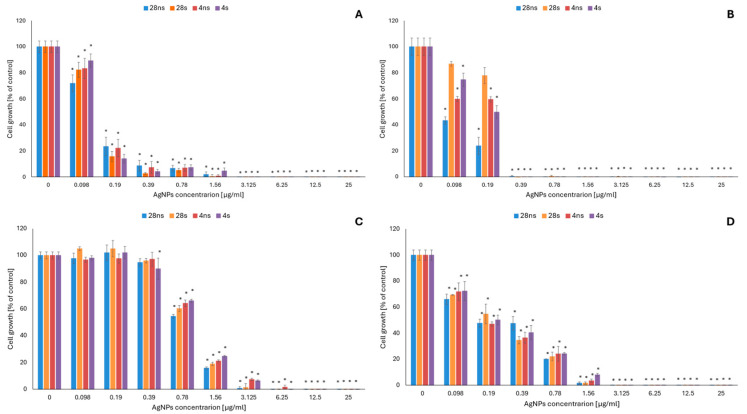
Antifungal activity of *G. striatum*-derived silver nanoparticles (AgNPs) against *C. albicans* (**A**), *M. furfur* (**B**), *A. flavus* (**C**) and *A. fumigatus* (**D**). The results are shown as average percentage values with standard deviations of the optical density (OD) of the biotic control. Statistical significance (*p* < 0.05) is shown by an asterisk (*).

The antifungal activity of *G. striatum*-derived AgNPs was determined at nanoparticle concentrations ranging from 0.098 to 25 μg/mL against four different human pathogenic fungal strains: the yeasts *C. albicans* ATCC 10231 and *M. furfur* DSM 6170 and the filamentous fungi *A. flavus* ATCC 9643 and *A. fumigatus* ATCC 204305. It was confirmed that, compared to the filamentous fungi, the tested yeast strains were more susceptible to the action of AgNPs (Figure 1).

Among all tested organisms, the *M. furfur* strain, with a minimal inhibitory concentration (MIC) of 0.39 μg/mL for all tested AgNP types, was found to be the most sensitive (Table 1). In *C. albicans*, growth inhibition reached 85% or higher using all tested AgNP types at a concentration of 0.39 μg/mL. However, according to the obtained MIC values (Table 1), the 4 s AgNP type was the least active against the tested microorganism—total growth inhibition was observed at a concentration of 3.125 μg/mL, while the same effect for the rest of the AgNPs tested was obtained using a two-fold lower concentration. Among the tested filamentous fungal strains, *A. fumigatus* was the most susceptible to the action of mycogenic AgNPs, with a 50% or higher inhibitory effect on cell growth obtained using all tested AgNP types at a concentration of 0.39 μg/mL (Figure 1). The MIC values obtained for this strain were 1.56 μg/mL for 28 ns and 28 s AgNPs and 3.125 μg/mL for 4 ns and 4 s AgNPs (Table 1). *A. flavus* was the least sensitive to the AgNPs. For this strain, the obtained MIC values were 3.125, 3.125, 6.25 and 12.5 μg/mL using 28 ns, 28 s, 4 s and 4 ns AgNPs, respectively (Table 1). Minimal fungicidal concentration (MFC) values (Table 1) were also determined in the current study and reached 25 μg/mL or exceeded the tested range of AgNP concentrations.

### 2.2. Changes in the Phospholipid Profiles of Fungal Cells in the Presence of Mycogenic AgNPs

**Figure 2:**
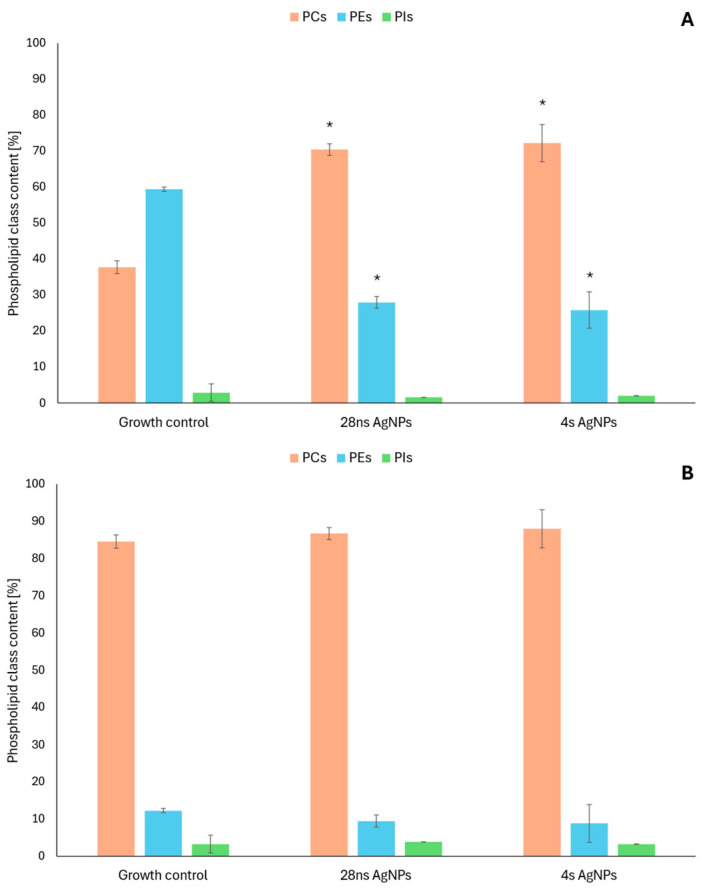
Changes in phospholipid content in the presence of 28 ns AgNPs and 4 s AgNPs: *A. flavus* (**A**) and *C. albicans* (**B**). The results are shown as average values with standard deviations. The statistical significance (*p* < 0.05) is shown by an asterisk (*).

To further characterize the antifungal activity of mycogenic AgNPs, changes in the phospholipid profiles of *C. albicans* and *A. flavus* were evaluated. To accomplish this task, the following experimental variations were selected based on the AgNP antifungal activity determined in the previous experiments—28 ns AgNPs and 4 s AgNPs at a concentration of 0.19 μg/mL for *C. albicans* and 1.56 μg/mL for *A. flavus*. As a result, three phospholipid classes were detected in each of the tested fungal strains—phosphatidylcholines (PCs), phosphatidylethanolamines (PEs) and phosphatidylinositols (PIs). In *C. albicans* (Figure 2B), visible changes were noted in the content of PCs and PEs. The levels of PCs increased and those of PEs slightly decreased in the presence of both tested AgNP types compared to the growth control. The PI content was barely affected by the AgNPs. In the case of *A. flavus* (Figure 2A), changes in the content of the same phospholipid classes were observed. The presence of 28 ns and 4 s AgNPs caused about a two-fold increase in the content of PCs compared to the growth control. In parallel, a significant decrease in PEs was observed—the content of this phospholipid class was reduced by more than 50%. The content of PIs was only slightly influenced by the tested AgNPs.

The PC/PE ratios and Double Bond Indexes (DBIs) were calculated and are shown in Table 2. In both *A. flavus* and *C. albicans*, the PC/PE ratio increased in the presence of the tested AgNPs, and 4 s AgNPs had a slightly stronger effect. Changes in the DBI induced by the presence of AgNPs were observed only in the case of *A. flavus*, in which the DBI increased. In the case of *C. albicans*, no changes were detected.

Specific results for certain phospholipid species detected in this study are shown in Tables S1 and S2 in the Supplementary Materials. In *C. albicans*, the most visible changes were observed in PC 16:0 18:2, PC 16:0 18:1 PC 18:3 18:2, PC 18:3 18:1, PC 18:0 18:2, PE 16:0 18:2 and PE 16:0 18:1. The contents of the mentioned PC and PE species decreased in the presence of the tested mycogenic AgNPs. In *A. flavus*, the most visible changes were detected in PC 16:0 18:2, PC 16:0 18:1, PC 18:2 18:2, PC 18:2 18:1, PC 18:1 18:1, PC 18:0 18:2, PE 16:0 18:2, PE 16:0 18:1, PE 18:2 18:1 and PE 18:1 18:1. Here, the content of PC and PE species with one saturated 16C acyl chain decreased in the presence of both tested AgNP types, by as much as 4 times in the case of PE 16:0 18:2 compared to the growth control. The levels of PCs and PEs with two unsaturated acyl chains increased in the same conditions, and the most significant change was observed in the case of PC 18:2 18:1 and PC 18:1 18:1, with a 6-fold or higher increase in the content of certain phospholipid species compared to the untreated samples.

### 2.3. Changes in the Cell Membrane Properties of C. albicans in the Presence of Mycogenic AgNPs

#### 2.3.1. Cell Membrane Fluidity

**Figure 3:**
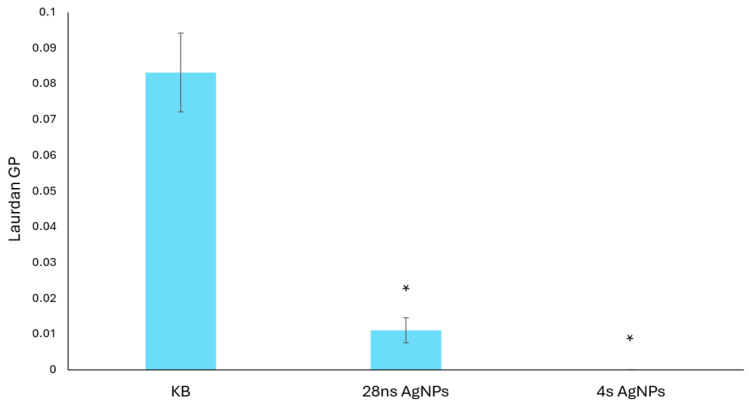
Changes in the cell membrane fluidity of *C. albicans* caused by the presence of AgNPs. The results are shown as average values with standard deviations of the calculated Laurdan GP parameter. The statistical significance (*p* < 0.05) is shown by an asterisk (*).

Changes in the cell membrane fluidity of *C. albicans* were determined in the presence of 28 ns and 4 s AgNPs at a concentration of 0.19 μg/mL using the Laurdan fluorescence spectroscopy method. The calculated generalized polarization (GP) parameter of Laurdan (Figure 3) confirmed that the presence of both tested AgNP types caused an increase in *C. albicans* cell membrane fluidity compared to the growth control. Moreover, it was observed that 4 s AgNPs at the tested concentration had a more severe effect.

#### 2.3.2. Cell Membrane Permeability

**Figure 4:**
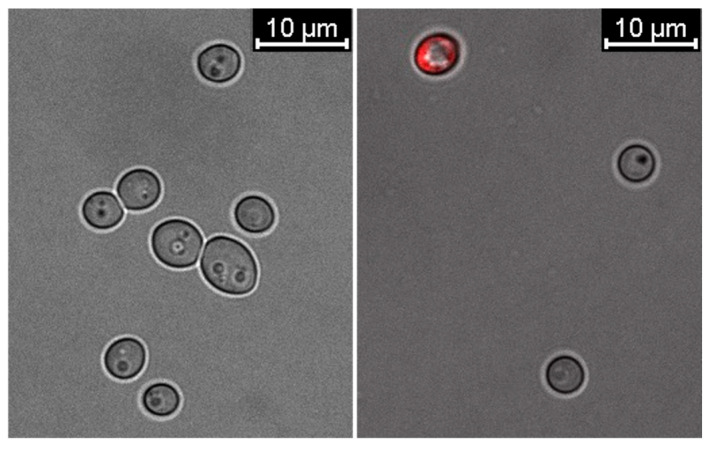
Changes in the cell membrane permeability of *C. albicans* in the presence of 0.19 μg/mL 28 ns AgNPs. The left image shows biotic control cells not treated with AgNPs. The right image shows cells treated with 28 ns AgNPs.

Confocal microscopy imaging of untreated *C. albicans* cells and those treated with 0.19 μg/mL 28 ns AgNPs was performed using propidium iodide staining. The analysis of the obtained images (Figure 4) revealed that the presence of the tested AgNP type caused a reduction in the growth and number of *C. albicans* cells compared to the untreated sample. Moreover, about 30% of the cells treated with AgNPs showed an increase in cell membrane permeability.

## 3. Discussion

Filamentous fungi have emerged as attractive candidates for AgNP synthesis. This is mostly due to their ease of cultivation, high metal tolerance and ability to secrete large amounts of extracellular agents that play a key role in silver ion reduction [32]. Fungi-derived AgNPs are proven to possess antibacterial properties. For instance, AgNPs synthesized using the endophytic fungus *Penicillium radiatolobatum* showed concentration-dependent activity against Gram-positive and Gram-negative bacterial strains [33]. In our previous study, AgNPs synthesized using *G. striatum* DSM 9592 strains in different synthesis conditions were found to be active against various bacterial strains [18]. Our current results prove that all obtained AgNP types also exhibit antifungal potential.

The antifungal activity of the synthesized AgNPs was tested against four fungal strains: *C. albicans*, *M. furfur*, *A. flavus* and *A. fumigatus*. The effectiveness of the AgNPs varied depending on the synthesis conditions. As stated previously, changes in the temperature and shaking conditions during the production process determined the physicochemical properties of the obtained AgNP types [18]. It is known that the antifungal performance of AgNPs is highly dependent on their characteristics, e.g., particle size [34]. Here, both types of AgNPs synthesized at 28 °C were more effective against almost all tested fungal strains compared to the other nanoparticles. Generally, yeast strains were more sensitive to the action of AgNPs compared to the filamentous fungal strains tested, and *C. albicans* was found to be more resistant than the other yeast strains, with 4-fold or 8-fold higher MIC values depending on the AgNP type. Barabadi et al. found that AgNPs synthesized using *Penicillium fimorum* were effective against *C. albicans* ATCC 10231, reaching a MIC value of 4 μg/mL [34], which indicated that *G. striatum*-derived AgNPs were more effective against that strain. A similar conclusion can be made for *M. furfur* investigated in another study. Sathishkumar et al. showed that biogenic AgNPs of plant origin were active against the microorganism, with a calculated MIC value of 25 μg/mL [35]. Compared to these results, the mycogenic AgNPs synthesized in our study were up to 64 times more active against *M. furfur* than those originating from plants. Among filamentous fungi, *A. flavus* was less sensitive to the action of all obtained AgNPs compared to the second tested strain. The opposite phenomenon was observed for AgNPs of bacterial origin synthesized using *Bacillus thuringiensis*. Here, *A. fumigatus* was observed to be more resistant to the action of AgNPs—the MIC value of the AgNPs against this strain was 62.5 μg/mL, while in the case of *A. flavus*, it reached 15.6 μg/mL [36]. Both values were higher than those revealed in our study, again indicating that the obtained AgNPs have stronger activity against the tested fungal strains. Our findings regarding the antifungal activity of mycogenic AgNPs are especially important in light of the results of a cytotoxic potential assessment of *G. striatum*-derived AgNPs obtained in our previous study [18]. According to these results, the obtained AgNPs are active against some of the tested fungal species at concentrations that caused less than a 50% decrease in the viability of a human fibroblast cell line. It can thus be concluded that AgNPs synthesized using *G. striatum* are active against the chosen pathogenic strains and simultaneously have no severe cytotoxic effects.

The MIC and MFC values obtained in our study suggest that *G. striatum*-derived AgNPs exhibited fungistatic rather than fungicidal properties. In another study, AgNPs obtained using green nanotechnology employing *Aspergillus terreus* acted similarly against *Aspergillus niger* strain. Here, the MFC value were 10 μg/mL while the obtained MIC value for this strain reached 0.312 μg/mL [37]. The same phenomenon was observed using plant-derived AgNPs and *C. albicans*, where the MFC values were at least 2-fold higher than the MICs obtained for nanoparticle types varying in size and reached 124.33 ± 4.04 µg/mL or more [38]. On the other hand, Różalska et al. found that AgNPs synthesized using *Metharizium robertsii* waste biomass showed stronger fungicidal activity against *C. albicans* ATCC 10231, with obtained MIC and MFC values of 1.56 μg/mL [39]. Our study indicates that, in terms of the calculated MIC values, all tested types of *G. striatum*-derived AgNPs were effective against *M. furfur*. Another study focusing on the efficacy of chemically synthesized AgNPs against *M. furfur* isolates showed that they had almost three-fold lower fungistatic activity against the tested strain compared to those tested in our study. However, the MFC values obtained in that study ranged between 0.5 and 2 mg/L, indicating that chemically produced AgNPs were more effective [40]. *M. furfur* is a lipid-dependent species, lacking the ability to perform de novo synthesis of fatty acids [41]. This fact could affect its ability to adapt its cell membrane properties to an inhospitable growth environment caused by the presence of AgNPs and might explain the increased susceptibility of this strain to the action of AgNPs compared to the other fungal species tested.

The antifungal potential of AgNPs has been attributed to their interference with fungal cell membrane integrity [42,43]. Phospholipids (PLs) constitute important structural and functional components of cell membranes. The exact composition of PLs can determine cell membrane properties, such as stability and fluidity. Thus, changes in the PL content can be considered a major indicator of perturbations to cell membrane functioning [44]. In our study, three PL classes were detected in both tested fungal species: PCs, PEs and PIs. It has been established that, in fungi, PCs and PEs are critical for the vegetative growth of mycelia [45]. PCs contribute to the formation and stabilization of the bilayer cell membrane form, while PEs are non-bilayer lipids forming single-layer phases, thus destabilizing the membrane structure [46]. In our study, an increase in the PC/PE ratio was observed in both tested microorganisms treated with AgNPs compared to the biotic control. This may indicate that the abiotic stress caused by the presence of AgNPs triggers phospholipid composition changes in the cell membrane, thus increasing its fluidity. One of the indicators that can be useful for evaluating changes in cell membrane properties is the Double Bond Index (DBI), which provides insights into membrane fluidity [47]. Generally, the occurrence of saturated fatty acids in PLs, consequently leading to the formation of straight acyl chains, decreases membrane fluidity due to the tight packing of these PLs in the membrane layers [48]. In our study, the DBI established for *A. flavus* increased in the presence of both types of AgNPs. It can be assumed that the presence of AgNPs altered the properties of the fungal cell membrane of *A. flavus*, increasing its fluidity. On the other hand, no differences in DBIs were obtained for untreated *C. albicans* cells or those treated with either of the tested AgNP types. However, the results of Laurdan fluorescence spectroscopy showed enhanced cell membrane fluidity in *C. albicans* cells in the presence of AgNPs, and the obtained effect was stronger when using 4 s AgNPs. The phenomenon was additionally confirmed by propidium iodide staining, where *C. albicans* cells showed increased cell membrane permeability in the presence of AgNPs. It cannot be excluded that AgNPs harm cells through multiple pathways, including a reduction in PE content, which was observed in *Pleurotus ostreatus* mycelia treated with cadmium ions [49]. Although the increase in the PC/PE ratio may suggest a more organized cell membrane state, it cannot be ruled out that other important components of the fungal cell membrane—sterols, sphingolipids and membrane proteins—contribute to the increased membrane fluidity observed. It is believed, based on many studies involving different species of yeast, that ergosterol plays a key role in regulating fungal membrane fluidity. Therefore, many antifungal drugs, including azoles, target this sterol [50].

Based on the obtained data, it can be concluded that AgNPs affect the structure of microbial biological membranes. However, further studies are required to explain the exact processes taking place.

## 4. Materials and Methods

### 4.1. Materials

The *C. albicans*, *A. flavus* and *A. fumigatus* strains were purchased from the American Type Culture Collection (ATCC, Manassas, Virginia, USA). The *M. furfur* strain was acquired from the German Collection of Microorganisms and Cell Cultures GmbH (Braunschweig, Germany). RPMI 1640 broth was purchased from Merck (Darmstadt, Germany). Sabouraud dextrose broth was obtained from Becton Dickinson (Warsaw, Poland). Glycerol and methanol came from Chempur (Piekary Slaskie, Poland). Chloroform and methanol of chromatographic purity, as well as propidium iodide and Laurdan stains, were obtained from Merck (Darmstadt, Germany).

#### AgNP Synthesis Using *Gloeophyllum striatum* DSM 9592

The AgNPs of mycological origin tested in this study were obtained using *Gloeophyllum striatum* DSM 9592 post-culture liquid; the synthesis process has been described previously. Four different nanoparticle variants were synthesized by using different temperatures and shaking conditions during the process, namely, 28 °C without shaking (28 ns AgNPs), 28 °C with shaking (28 s AgNPs), 4 °C without shaking (4 ns AgNPs) and 4 °C with shaking (4 s AgNPs). The obtained AgNPs were then separated from the *G. striatum* post-culture liquid by centrifugation and dispersed in sterile deionized water. Then, the synthesized AgNPs were analyzed in order to evaluate differences in their physicochemical properties depending on the synthesis scheme. All the synthesized AgNP types were confirmed to be polydispersed, and those produced without shaking were smaller than the other types when comparing the diameter sizes of the most numerous nanoparticle fractions. Moreover, other nanoparticle properties also differed, such as zeta-potential, which was lower in both nanoparticle types synthesized at 4 °C compared to those synthesized at 28 °C [18]. The prepared AgNPs were then used in further experiments.

### 4.2. Methods

#### 4.2.1. Evaluation of the Antifungal Activity of Mycogenic AgNPs

The antifungal potential of *G. striatum*-derived AgNPs was tested using the microdilution method in accordance with the Clinical and Standard Laboratory Institute (CSLI) guidelines M27 (4th Edition) for yeast strains and M38 (3rd Edition) for filamentous fungal strains. The antifungal activity was evaluated against four fungal strains, namely, *Candida albicans* ATCC 10231, *Malassezia furfur* DSM 6170, *Aspergillus flavus* ATCC 9643 and *Aspergillus fumigatus* ATCC 204305. The evaluation was performed in 96-well cell culture plates using different liquid media—RPMI 1640 broth for *C. albicans*, RPMI 1640 broth supplemented with glycerol for *M. furfur* and Sabouraud dextrose broth for filamentous fungal strains. The tested AgNPs were diluted in appropriate media to reach the final concentration range of 0.098–25 μg/mL. The inocula of the tested microorganisms were prepared in appropriate media, reaching a final density of 2.5 × 10^5^ spores/mL for filamentous fungi and 1 × 10^5^ colony-forming units (CFU)/mL for yeasts. Then, 96-well plates containing the samples and accordingly prepared abiotic and biotic controls were incubated for 48 h at 37 °C. After incubation, the optical density (OD) was measured at a wavelength of 630 nm using a Multiscan^TM^ FC Microplate Photometer (ThermoFisher Scientific, Pudong, Shanghai, China), and MIC values were determined for all experimental variants, with MIC defined as the lowest concentration of AgNPs at which no microorganism growth was observed in the plate wells. Then, the ZT plates were inoculated with 100 μL of fungal suspension samples with AgNP concentrations equal to the MIC value or higher in order to determine MFC values. The plates were then incubated for 48 h at 37 °C. MFC values were defined as the lowest concentration of AgNPs that reduced the viability of the tested fungal strains to zero, with no growth of colonies observed. Both MIC and MFC values were expressed in μg/mL.

#### 4.2.2. Changes in the Phospholipid Profiles of Fungal Cells in the Presence of Mycogenic AgNPs

Changes in the phospholipid profiles were analyzed in selected experimental variants based on the obtained results of antifungal activity. Specifically, 28 ns AgNPs and 4 s AgNPs were selected, and the evaluation was performed using two fungal strains: *A. flavus* and *C. albicans*. The tested concentrations of AgNPs varied depending on the tested strain and were 1.56 μg/mL and 0.19 μg/mL, respectively. Fungal cultures, including biotic and abiotic controls, were cultivated in the conditions described in Section 4.2.1. in a volume of 20 mL per sample. After incubation, the samples were centrifuged at 20 °C/5 min/8000 rpm. Then, supernatants were removed, and 8 mL of methanol was added to each sample. The prepared samples were homogenized using the ultrasound method. After homogenization, 16 mL of chloroform of chromatographic purity and 1 mL of 0.85% NaCl were added to each sample for the extraction process. Next, the organic phases were collected, evaporated and dissolved in methanol of chromatographic purity. The samples were then subjected to analysis.

The phospholipid analysis procedure was conducted as in Bernat et al. using an ExionLC AC UHPLC system (Sciex, Framingham, Massachusetts, USA) and a 4500 Q-TRAP mass spectrometer (Sciex, Framingham, Massachusetts, USA) with an ESI source at a spray voltage of −4.500 V and a temperature of 600 °C. A 10 μL volume of the lipid extract was injected onto a Kinetex C18 column (50 mm × 2.1 mm, particle size: 5 μm; Phenomenex, Torrance, California, USA) with a flow rate of 500 µL min^−1^ at 40 °C. The mobile phases, water (A) and methanol (B), contained 5 mM ammonium formate. The solvent gradient was initiated at 70% B, increased to 95% B over 1.25 min, and maintained at 95% B for 5 min before returning to the initial solvent composition over 2 min [51].

#### 4.2.3. Changes in *C. albicans* Cell Membrane Fluidity in the Presence of Mycogenic AgNPs

Changes in *C. albicans* cell membrane fluidity in the presence of mycogenic AgNPs were investigated with the use of the Laurdan fluorescence spectroscopy method. To accomplish this, *C. albicans* cells were cultivated in Sabouraud dextrose broth with and without 28 ns AgNPs and 4 ns AgNPs at a concentration of 0.19 μg/mL to reach an OD at a wavelength of 630 nm equal to 0.5. Next, suspensions of *C. albicans* cells cultivated with or without the supplementation of nanoparticles were transferred to different 2 mL reaction tubes. The Laurdan stain (6-Dodecanoyl-2-Dimethylaminonaphthalene) was then added to each tube to reach a final concentration of 10 μM. The prepared samples were incubated in the dark at 37 °C for 5 min. After incubation, the cells were washed four times with PBS and again adjusted to an OD_630_ of 0.5 in a volume of 2 mL. Then, 500 μL was transferred from each sample to a new reaction sample and centrifuged to obtain the supernatant serving as Laurdan background fluorescence. Then, 150 μL of each cell suspension sample and fluorescence background sample were transferred to a black flat-bottom 96-well microtiter plate. Laurdan fluorescence was measured using a SpectraMax i3 Multimode Microplate Reader (Molecular Devices, San Jose, California, USA) at an excitation of 350 nm and two emission wavelengths: 435 nm and 500 nm. The results are shown as the values of Laurdan generalized polarization (*GP*) parameters calculated according to the following formula:

$$GP=\frac{I_{435}-I_{500}}{I_{435}+I_{500}}$$

#### 4.2.4. Confocal Microscopy Analysis

*C. albicans* cells were cultivated as described in Section 4.2.1. with and without the addition of 0.19 μg/mL 28 ns AgNPs. Then, 1600 μL of each treated or untreated cell suspension was transferred to a new reaction tube, washed three times using PBS and stained with 3 mM propidium iodide. The samples were incubated in the dark for 15 min at room temperature. After incubation, the samples were again washed three times with PBS. Finally, 20 μL of each cell suspension was transferred to a microscopic slide and analyzed using a Leica TCS SP8 microscope equipped with plan achromatic objectives (Leica, Nußloch, Germany) with a magnification of 100× (oil immersion).

#### 4.2.5. Statistical Analysis

Every experiment presented in this study, excluding the confocal microscopy analysis, was performed in four replicates (*n* = 4). The results of the experiments were analyzed using a one-way ANOVA test with * *p* < 0.05 in order to estimate the statistical significance. The estimations and calculations were carried out by using Excel, Microsoft^®^ Office 2021 (Microsoft Corporation, Redmont, WA, USA). The results shown in the tables and figures are expressed as average values with standard deviations (SDs).

## 5. Conclusions

Our study revealed that AgNPs synthesized extracellularly using the post-culture liquid of *G. striatum* DSM 9592 have fungistatic potential. Among the tested fungal strains, *M. furfur* was found to be the most susceptible to the action of AgNPs. Changes in the phospholipid profiles of *C. albicans* and *A. flavus* caused by the presence of AgNPs were also analyzed in order to deduce a possible mechanism of nanoparticle action. It was found that in both tested species, the presence of AgNPs caused an increase in the PC/PE ratio. Based on the obtained DBIs, in *A. flavus*, the presence of AgNPs caused an increase in the content of unsaturated fatty acids, which may have been associated with enhanced cell membrane fluidity in response to AgNP treatment. In *C. albicans*, no changes in the DBI were observed. However, the results of Laurdan spectroscopy and confocal microscopy analyses confirmed that the cell membrane fluidity of *C. albicans* also increased in the presence of AgNPs. This finding may suggest that the properties of the cell membrane can be adjusted not only by changes in fatty acid saturation but also by other alterations in the membrane composition. Simultaneously, the versatility of mycogenic AgNPs originating from the brown-rot decay fungus was confirmed.

## Figures and Tables

**Table 1:** Minimum inhibitory and minimum fungicidal concentration (MIC and MFC, respectively, μg/mL) values of mycogenic AgNPs against selected fungal strains.

	MIC				MFC			
	28 ns	28 s	4 ns	4 s	28 ns	28 s	4 ns	4 s
*A. fumigatus* ATCC 204305	1.56	1.56	3.125	3.125	>25	>25	>25	>25
*A. flavus* ATCC 9643	3.125	3.125	6.25	12.5	>25	>25	>25	>25
*C. albicans* ATCC 10231	1.56	1.56	1.56	3.125	>25	>25	>25	25
*M. furfur* DSM 6170	0.39	0.39	0.39	0.39	>25	>25	>25	>25

**Table 2:** PC/PE ratios and DBIs established for *A. flavus* and *C. albicans* with and without treatment with 28 ns AgNPs and 4 s AgNPs.

	*A. flavus* ATCC 9643			*C. albicans* ATCC 10231		
	Growth Control	28 ns AgNPs	4 s AgNPs	Growth Control	28 ns AgNPs	4 s AgNPs
PC/PE	0.64	2.52	2.87	6.92	9.24	10.09
DBI	0.95	1.20	1.17	1.08	1.10	1.07

## Data Availability

The raw data supporting the conclusions of this article will be made available by the authors on request.

## Supplementary Materials

The following supporting information can be downloaded: https://www.mdpi.com/article/10.3390/ijms26146639/s1. Table S1: The influence of 28 ns and 4 s AgNPs at a concentration of 1.56 μg/mL on the phospholipid profile of *A. flavus* ATCC 9643. The asterisk * (*p* < 0.05) indicates values that differ significantly from the control. Table S2: The influence of 28 ns and 4 s AgNPs at a concentration of 0.19 μg/mL on the phospholipid profile of *C. albicans* ATCC 10231. The asterisk * (*p* < 0.05) indicates values that differ significantly from the control.
