# Supplementary material for: Mycogenic Silver Nanoparticles: Promising Antimicrobials with Fungistatic Properties
Source: Int J Mol Sci. 2025 Jul 10;26(14):6639. doi: 10.3390/ijms26146639 (PMC12294507; doi:10.3390/ijms26146639)
Supplement: Supplementary file 1 [file ijms-26-06639-s001.zip › ijms-3707919-supplementary.pdf]

Tab. S1. The influence of 28ns and 4s AgNPs in the concentration of 1.56 μg/ml on phospholipid profile of *A. flavus* ATCC 9643. Asterisk * ($p < 0.05$) indicates values that differ significantly from the control.

Phospholipid species	Growth control	28ns AgNPs	4s AgNPs
PC 16:0 18:3	0.727±0.84	0.013±0.00	0.011±0.00
PC 16:0 18:2	12.060±1.02	**5.021±0.20***	**5.469±0.60***
PC 16:0 18:1	10.656±3.24	5.299±0.10	6.793±0.91
PC 18:3 18:3	0.003±0.00	0.004±0.00	0.008±0.01
PC 18:3 18:2	0.034±0.03	0.108±0.03	0.123±0.04
PC 18:3 18:1	0.018±0.01	0.101±0.04	0.086±0.03
PC 18:2 18:2	4.646±1.60	**15.072±0.29***	**14.580±1.13***
PC 18:2 18:1	2.953±0.03	**19.989±0.76***	**18.626±2.07***
PC 18:1 18:1	3.921±0.50	**24.623±0.92***	**26.319±2.67***
PC 18:0 18:2	2.723±0.13	**0.184±0.03***	**0.193±0.04***
PE 16:1 16:1	0.064±0.04	0.046±0.01	0.024±0.01
PE 16:1 18:2	0.440±0.20	0.334±0.02	0.279±0.01
PE 16:0 18:3	0.391±0.05	0.242±0.07	**0.240±0.01***
PE 16:0 18:2	41.819±1.43	**11.437±0.86***	**9.155±2.57***
PE 16:0 18:1	12.287±0.03	**7.175±0.23***	**7.932±1.42***
PE 18:3 18:2	0.031±0.03	0.004±0.00	0.007±0.01
PE 18:3 18:1	0.012±0.01	0.009±0.00	0.004±0.00
PE 18:2 18:2	1.247±0.48	1.331±0.11	0.972±0.08
PE 18:2 18:1	1.264±0.18	**2.559±0.06***	**2.114±0.18***
PE 18:1 18:1	1.810±0.04	**4.818±0.27***	**5.082±0.81***
PI 16:0 18:2	1.322±1.30	0.484±0.08	0.507±0.02
PI 16:0 18:1	1.572±1.08	1.145±0.09	1.476±0.05

Tab. S2. The influence of 28ns and 4s AgNPs in the concentration of 0.19 µg/ml on phospholipid profile of *C. albicans* ATCC 10231. Asterisk * ($p < 0.05$) indicates values that differ significantly from the control.

Phospholipid species	Growth control	28ns AgNPs	4s AgNPs
PC 16:0 18:3	0.019±0.00	0.017±0.01	0.020±0.00
PC 16:0 18:2	6.510±0.02	4.732±0.75	**3.869±0.01***
PC 16:0 18:1	12.376±0.14	**10.723±0.27***	**10.280±0.33***
PC 18:3 18:3	0.000±0.00	0.002±0.00	0.000±0.00
PC 18:3 18:2	0.286±0.00	**0.089±0.03***	**0.088±0.02***
PC 18:3 18:1	0.547±0.03	**0.209±0.01***	**0.168±0.02***
PC 18:2 18:2	6.830±0.11	6.972±2.11	**5.395±0.00***
PC 18:2 18:1	13.722±0.37	16.560±2.33	13.762±0.98
PC 18:1 18:1	42.785±0.12	46.543±4.79	**53.595±2.08***
PC 18:0 18:2	1.459±0.11	**0.867±0.00***	**0.790±0.03***
PE 16:1 16:1	0.808±0.08	**1.459±0.03***	1.026±0.18
PE 16:1 18:2	1.188±0.04	1.212±0.07	0.993±0.15
PE 16:0 18:3	0.004±0.00	0.006±0.00	0.005±0.00
PE 16:0 18:2	2.757±0.21	1.628±0.39	**1.389±0.22***
PE 16:0 18:1	1.510±0.09	**0.774±0.08***	**0.842±0.06***
PE 18:3 18:2	0.001±0.00	0.002±0.00	0.001±0.00
PE 18:3 18:1	0.031±0.00	**0.005±0.00***	**0.004±0.00***
PE 18:2 18:2	0.873±0.13	0.608±0.04	**0.430±0.06***
PE 18:2 18:1	1.793±0.04	1.203±0.33	**1.114±0.18***
PE 18:1 18:1	3.261±0.08	2.541±0.26	3.013±0.39
PI 16:0 18:2	0.854±0.02	1.041±0.15	0.781±0.14
PI 16:0 18:1	2.386±0.20	2.808±0.12	2.435±0.02
